# THE EFFECTS OF FRAILTY, POLYPHARMACY, AND COGNITION ON HEALTH OUTCOMES: A STUDY ON INTERRAI RESIDENTIAL CARE DATA

**DOI:** 10.1093/geroni/igz038.330

**Published:** 2019-11-08

**Authors:** Macy Zou, Ronald Kelly, Betty Chinda, Mckenzie Braley, Tony Zhang, Tara Arvan, Robert Mcdermid, Xiaowei Song

**Affiliations:** 1 Health Research and Innovation, Surrey Memorial Hospital, Fraser Health Authority, Surrey, British Columbia, Canada; 2 interRAI Evaluation & Research, Fraser Health Authority, Surrey, British Columbia, Canada; 3 Department of Emergency Medicine, Surrey Memorial Hospital, Fraser Health Authority, Surrey, British Columbia, Canada

## Abstract

Frailty Index (FI), polypharmacy and cognition status are significant health concerns in older adults. We conducted this study to investigate the interplay of frailty, polypharmacy, and cognition, in determining health outcomes. InterRAI Residential Care (RAI-RC MDS2.0) data were retrieved from residential care homes in Surrey, BC, Canada. Older residents (65+ years) who had RAI-RC records between 2016 and 2018 were used in the analysis (n=976). A deficit accumulation-based FI was generated using 36 variables. Information on polypharmacy and cognition were obtained by accounting the total number of medications and the cognitive performance scale. Information on falls, emergency visits, and mortality were followed. Multivariate Cox proportional hazard models were used to examine the effects of these variables on different outcomes. The FI showed a near Gaussian distribution (median= 0.370 mean= 0.372 SD= 0.143), and increased linearly with age on a logarithm scale (R=0.75, p<0.001). Residents with cognitive impairment showed a higher level of the FI (KW= 863.3, p<0.001). A higher FI was associated with an increased risk of death (HR=15.2 p=0.006) and emergency visits (HR=2.72 p=0.048), adjusting for age, sex, medications, and education levels. Frailty, polypharmacy, and cognition levels are associated and have interactive effects on health outcomes. Ongoing research is to validate the findings with large samples in different health settings, and to understand the underlying processes of the effect. The close relationships between frailty, polypharmacy, and cognition with health outcomes call for effective integrated strategies for healthcare of older adults with multiple complex health problems.

